# Mass Spectrometry Imaging and Integration with Other Imaging Modalities for Greater Molecular Understanding of Biological Tissues

**DOI:** 10.1007/s11307-018-1267-y

**Published:** 2018-08-30

**Authors:** Tiffany Porta Siegel, Gregory Hamm, Josephine Bunch, Jo Cappell, John S. Fletcher, Kristina Schwamborn

**Affiliations:** 1 Maastricht Multimodal Molecular Imaging (M4I) Institute, Division of Imaging Mass Spectrometry, Maastricht University, Maastricht, The Netherlands; 2 Astrazeneca IMED-DSM, Cambridge, UK; 3 NPL Teddington, Teddington, UK; 4 Department of Chemistry and Molecular Biology, University of Gothenburg, Gothenburg, Sweden; 5 Technische Universität München, Institut für Allgemeine Pathologie und Pathologische Anatomie, Munich, Germany

**Keywords:** Mass spectrometry imaging (MSI), Molecular imaging, Imaging mass cytometry (IMC), Magnetic resonance imaging (MRI), Histology, Pathology

## Abstract

Over the last two decades, mass spectrometry imaging (MSI) has been increasingly employed to investigate the spatial distribution of a wide variety of molecules in complex biological samples. MSI has demonstrated its potential in numerous applications from drug discovery, disease state evaluation through proteomic and/or metabolomic studies. Significant technological and methodological advancements have addressed natural limitations of the techniques, *i.e.*, increased spatial resolution, increased detection sensitivity especially for large molecules, higher throughput analysis and data management. One of the next major evolutions of MSI is linked to the introduction of imaging mass cytometry (IMC). IMC is a multiplexed method for tissue phenotyping, imaging signalling pathway or cell marker assessment, at sub-cellular resolution (1 μm). It uses MSI to simultaneously detect and quantify up to 30 different antibodies within a tissue section. The combination of MSI with other molecular imaging techniques can also provide highly relevant complementary information to explore new scientific fields. Traditionally, classical histology (especially haematoxylin and eosin–stained sections) is overlaid with molecular profiles obtained by MSI. Thus, MSI-based molecular histology provides a snapshot of a tissue microenvironment and enables the correlation of drugs, metabolites, lipids, peptides or proteins with histological/pathological features or tissue substructures. Recently, many examples combining MSI with other imaging modalities such as fluorescence, confocal Raman spectroscopy and MRI have emerged. For instance, brain pathophysiology has been studied using both MRI and MSI, establishing correlations between in and *ex vivo* molecular imaging techniques. Endogenous metabolite and small peptide modulation were evaluated depending on disease state. Here, we review advanced ‘hot topics’ in MSI development and explore the combination of MSI with established molecular imaging techniques to improve our understanding of biological and pathophysiological processes.

## Introduction to Mass Spectrometry Imaging (MSI)

Knowledge of the regional distribution of molecular species is essential to understand biological processes occurring within tissues. This information is traditionally lost through using liquid chromatography coupled to mass spectrometry (LC-MS) analysis of tissue homogenates. In contrast, for mass spectrometry imaging (MSI), intact tissue sections are analysed. Thus, this technology can be used to spatially resolve the distribution of endogenous (small metabolites, lipids, peptides and proteins) and exogenous (drugs and drug metabolites) species in tissue sections.

**Fig. 1:**
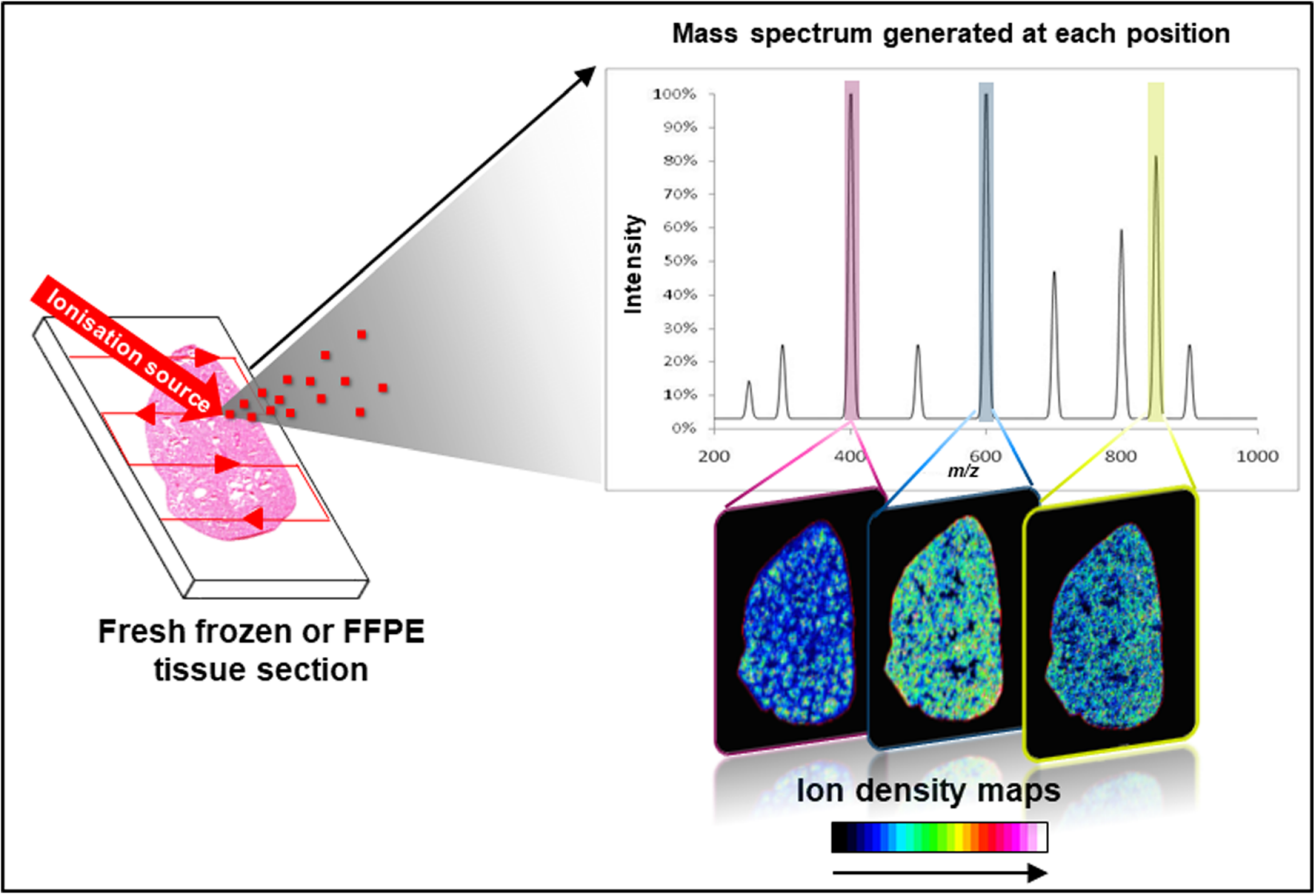
Mass spectrometry imaging (MSI) workflow.

**Fig. 2:**
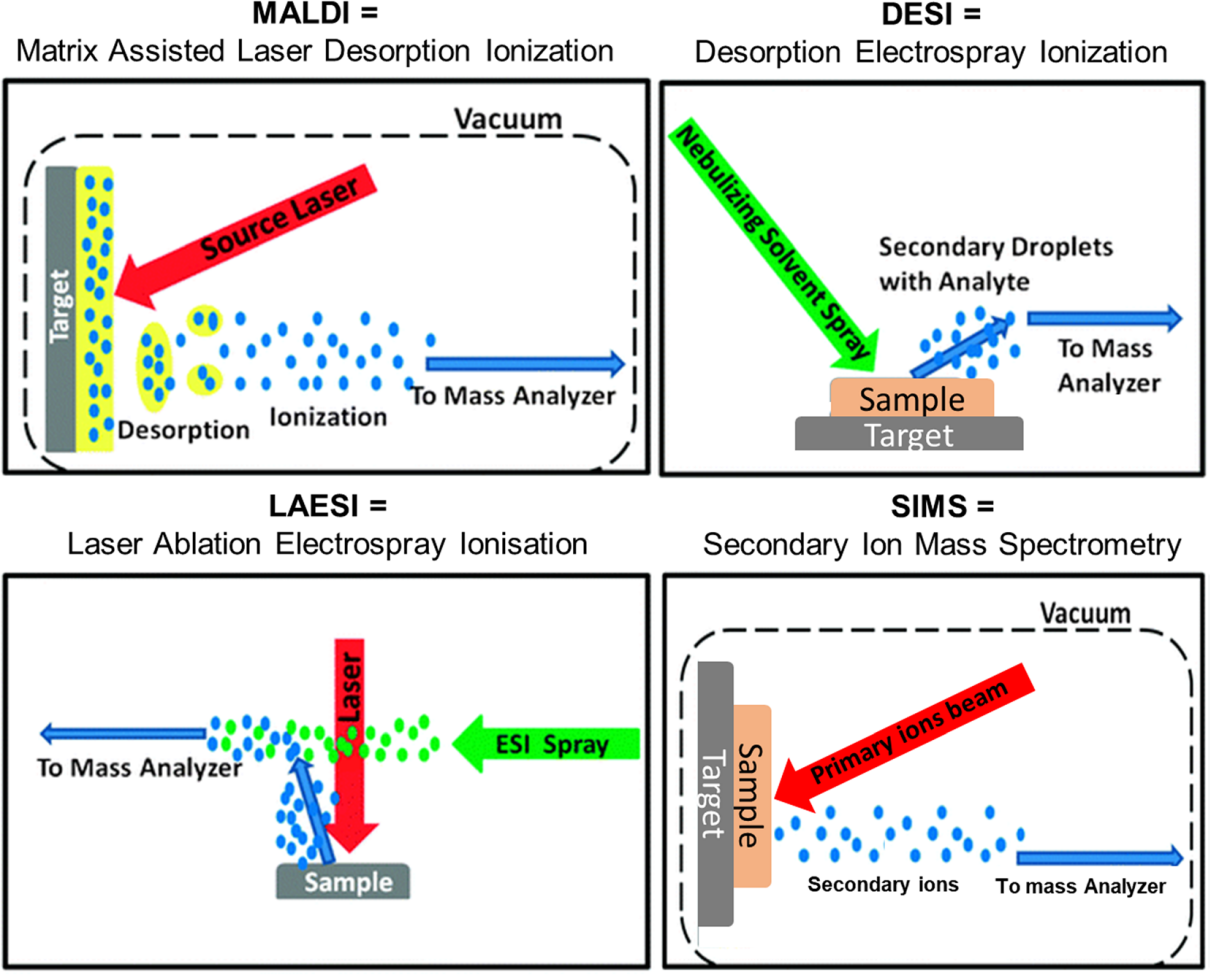
Comparison of main ionisation techniques employed for MSI.

Fig. 1 presents the workflow of MSI. Originally introduced in 1997 by Caprioli *et al*. [1], a label-free ionisation technique rasters a biological sample (fresh frozen or formalin-fixed paraffin-embedded (FFPE) tissue section) in order to generate charged species that are analysed by a mass spectrometer. For each position of the virtual raster pattern, a mass spectrum is generated, reporting the intensities at thousands of mass-to-charge (*m*/*z*) ratios that relate to specific molecular species. An ion density map can then be generated for each *m*/*z* value detected which visualises their spatial localisation as well as relative intensity. Matrix-assisted laser desorption/ionisation (MALDI) [1], desorption electrospray ionisation (DESI) [2], laser ablation electrospray ionisation (LAESI) [3] and secondary ion mass spectrometry (SIMS) are the main ionisation techniques used for mass spectrometry imaging measurements. These are described in Fig. 2. Each of these ionisation techniques can be combined with different mass analysers to access different spectral resolutions, dynamic ranges or throughput; for example, time-of-flight (ToF), Fourier transform ion cyclotron resonance (FTICR), Orbitrap or triple quadrupole (QQQ) mass analysers. These combinations offer the ability to detect different molecular classes, improve sensitivity, enhance the specificity, give a higher spatial resolution and/or faster measurement.

MSI has already demonstrated enormous potential for biomedical research and pharmaceutical applications. It enables untargeted as well as direct targeted analysis to discover disease-related biomarkers. Thus, prognostic and diagnostic studies of disease like cancer have been performed that are based on the modulation of endogenous species spatially correlated with pathological features in tissues [4]. In pharmaceutical research, MSI is applied in both preclinical and clinical studies to evaluate drug delivery and assess compound safety, efficacy and target interaction [5]. Quantification of small molecules has recently gained interest and numerous methods have been developed to tackle the challenge of tissue suppression normalisation [6, 7]. Thus, MSI is an attractive tool for potential combination with other molecular imaging techniques enabling multimodality and benefitting from information gained by other imaging modalities.

Here, we highlight the potential of MSI and recent developments in the field. First, we illustrate the combination of MSI with histology for histopathological evaluation of cancer tissue for both enhanced molecular understanding of cancer biology and diagnostic capabilities. Secondly, we explore the limits of MSI in terms of ultra-high spatial resolution imaging for single cell analysis and capabilities of targeted, multiplexed antibody imaging using imaging mass cytometry (IMC). Finally, we discuss the largest challenges the MSI community is facing regarding the interpretation and analysis of big data as well as advances being made for its integration with other *in vivo* molecular imaging modalities.

### Combining MSI with Histology for Histopathological Evaluation of Cancer Tissue

Nowadays, cancer pathology is no longer a simple evaluation of tissue specimens using basic stains such as haematoxylin and eosin (H&E) in order to come to a diagnosis of cancer. Many more subtypes of cancer have been discovered, some only based on molecular differences. Additionally, many drug-able targets have been identified in different cancers that can only be detected using antibodies or molecular tests. Thus, pathology has embraced techniques such as immunohistochemistry (IHC) and fluorescence *in situ* hybridisation (FISH), as well as extraction-based molecular diagnostics such as gene sequencing, in order to incorporate all findings together with classical histomorphology. This research area is now known as molecular histology. It is intended to reveal the underlying biochemistry of tissues and organs, while simultaneously providing information on the influence of therapeutics or toxins on the function or misfunction of an organ [8]. Tissues have an inherent heterogeneity that increases further in diseases like cancer. Therefore, techniques that can measure the relative abundance of different types of molecule in a spatially resolved manner without the need for microdissection or target-specific reagents are ideal for molecular histology.

In the field of molecular histology, many studies have focused on oncological research in order to find new diagnostic, prognostic or predictive markers in different types of cancer that can indicate more personalised effective therapy regimes (reviewed elsewhere [4]). In a recent study, MALDI-MSI achieved reliable entity subtyping in non-small cell lung cancer [9]. Utilising tissue microarrays (TMA) with a total of 326 patient samples (168 with primary adenocarcinomas and 158 primary squamous cell carcinomas of the lung) and a linear discriminant analysis–based model, all but one case were accurately classified. Another study used a previously established classification method and algorithm to classify 102 atypical Spitzoid neoplasms. These are difficult to diagnose, ambiguous lesions in the borderline area between Spitz nevi and Spitzoid melanoma, sourced from 11 different countries [10]. Overall, the MS-based diagnosis (Spitz nevi or Spitzoid melanoma) showed a stronger association with clinical outcome compared to the histopathological diagnosis.

**Fig. 3:**
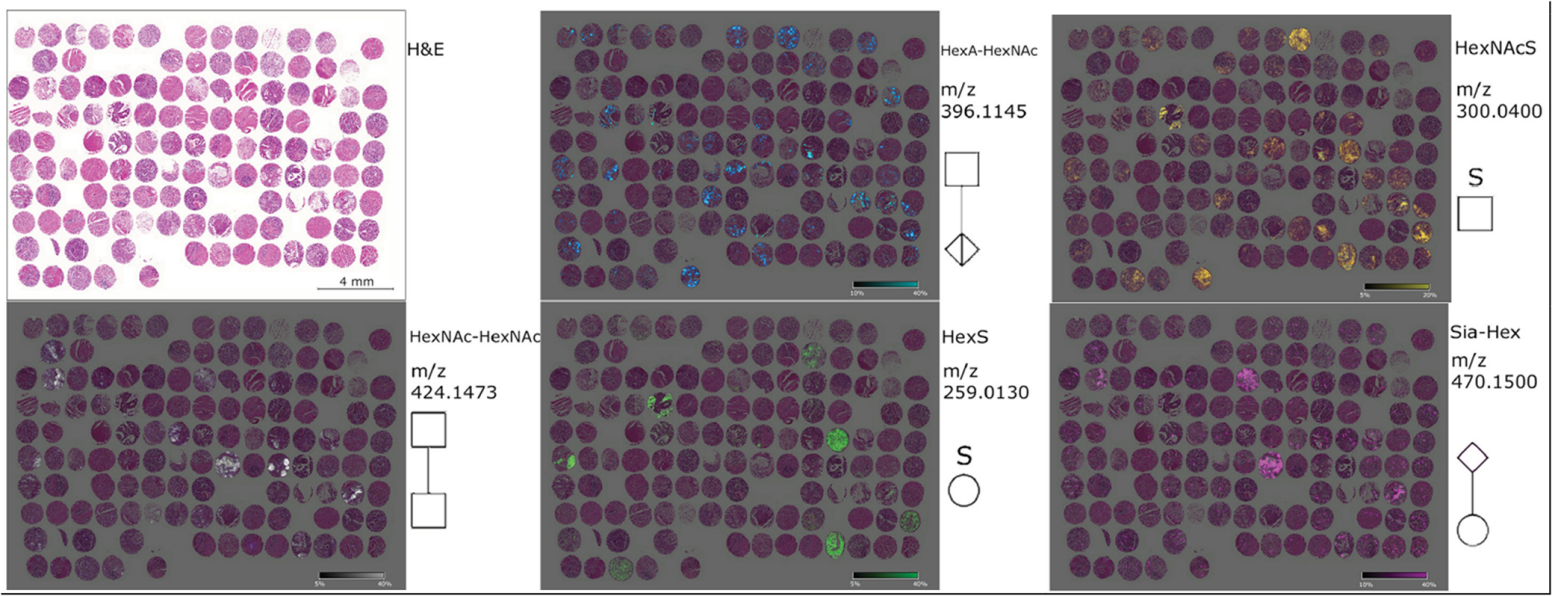
Gastric cancer TMA sample analysed by MSI showing the distribution of five native glycan fragments (HexNAc-HexNAc, HexNAcS, HexA-HexAc, HexS and Sia-hex). These glycans are specific for tissue compartments according to histological overview from H&E image and patients’ prognosis. Reproduced from [13].

Another possible application for MSI in the field of oncology is the identification of prognostic signatures beyond classical histology. Lou *et al*. could identify proteins and protein isoforms that were associated with patient survival in four different high-grade sarcoma subtypes [11]. High expression of proteasome activator complex subunit 1 (PSME1, *m*/*z* 9753) in leiomyosarcoma (*n* = 12), myxofibrosarcoma (*n* = 13) and undifferentiated pleomorphic sarcoma (*n* = 12) was associated with poor survival. In colorectal adenocarcinoma, 15 *m*/*z* values were identified as potential prognostic markers utilising a TMA containing samples from 349 patients [12]. Multivariate analysis revealed five out of these *m*/*z* values to have an independent prognostic role. Combining them in a score based on presence (weak and/or strong intensity) or absence of the *m*/*z* signal provided prognostic relevance independently from tumour and nodal stage. Fig. 3 displays the distribution of five native glycan fragments in gastric cancer TMA sample analysed by MSI. Glycans have also been utilised for prognostic analysis in cancers. In gastric adenocarcinoma (*n* = 106), five glycans were linked to patient prognosis [13]. Multivariate statistical analysis revealed one of these (HexNAc–HexA–HexNAc) as an independent prognostic factor.

In many biomarker studies, IHC is used as a tool to independently verify mass spectrometric findings. However, it can also be used to guide and better understand MSI results as has been shown by Huber *et al*. [14]. Through the combination of vasculature staining (using an anti-CD31 antibody) co-registered with mass spectrometric imaging, a higher degree of vascularisation as well as vessel characteristics could be linked to high drug levels within tumour regions. Depending on the heterogeneity of the tissue, staining consecutive sections is sometimes not an option as small features of interest (single cells and small groups of cells) are not consistent between serial sections. Thus, efforts have been made to reuse the section previously analysed by imaging techniques such as MALDI or DESI for staining. Regarding classical stains, in particular H&E, this process is now well established without significant loss of histological information or quality compared to stained serial sections. IHC and immunofluorescence staining following imaging experiments has proven more difficult [15] but is necessary in many instances to identify cell types or tissue features of interest. Regarding MALDI, choosing a matrix that requires less laser energy for molecule ionisation can minimise tissue distortion and epitope degradation through laser ablation. This strategy has been shown to be successful in correlating ion images and fluorescent microscopy images to identify and characterise amyloid aggregates [16].

### Ultra-High Spatial Resolution Imaging Using SIMS Combined with Surface Analysis

Secondary ion mass spectrometry (SIMS) uses a beam of energetic (typically tens of kiloelectron volts) *primary ions* to ablate material, some of which is ionised to form *secondary ions*, through a process called sputtering. SIMS is one of the oldest MS imaging modalities with examples of biological imaging dating to the 1960s [17, 18]. For many years, SIMS was predominantly employed in the semiconductor industry for measuring dopant levels in silicon. Despite being closely linked with the development of fast atom bombardment (FAB)-MS, forerunner to MALDI-MS, the SIMS community drifted away from general mass spectrometry during the 1980s and 1990s. While MALDI revolutionised bio-MS and was adapted for imaging, SIMS imaging was mainly being applied to studies related to catalysis and materials surface characterisation. Here, the very high surface sensitivity of SIMS (secondary ions originate from the outer few nanometres of the sample surface) was an advantage. The lateral resolution possible with SIMS normally depends on the ability to focus the primary beam onto the sample surface (microprobe SIMS) although instruments have been developed that also operate with defocused ion beams with position-sensitive detectors (microscope mode) [19, 20].

Different types of primary ion beams can be easier or more difficult to focus and influence the number and type of the secondary species that are ejected. Recently, a modified He-ion microscope has been used to perform SIMS with *ca.* 10 nm lateral resolution. Normally, SIMS images however range from tens of nanometre to several micrometre resolution and the secondary species that are generated depend heavily on the type of primary ion that is fired at the sample. Different SIMS instruments are available with different ion beam and mass analyser combinations that have particular strengths and weaknesses depending on the application area [21]. By operating a Cs^+^ ion beam in a coaxial primary/secondary ion optical column allowing a very short working distance for the ionoptical lenses, the ‘NanoSIMS’ instruments manufactured by Cameca can deliver MS images with 50 nm lateral resolution. However, analysis using monoatomic ion beams, such as Cs^+^, at high primary ion fluence limits the experiment to the detection of elements or very small (*e.g.*, diatomic) fragments of molecules and the magnetic sector mass analyser on the current generation of this instrument can only detect seven species in parallel. Despite this limitation, the NanoSIMS has been used to great effect in a number of biological studies, where isotopic labelling has been used to follow proteins or metabolites of neurotransmitters [22]. Metallic ions can also provide good targets for NanoSIMS analysis such as in the study of cisplatin distribution. Other studies have attached these to antibodies in mass cytometry studies—although as the NanoSIMS is only capable of imaging seven species at once multiplexing capability is reduced [23, 24]. For many years, the major drawback of SIMS as a bio-analytical tool was the limited ability of the ion beam–induced sputtering process to generate intact molecular ions above 1–200 Da. Recently, ion beam developments have started to overcome this with the atomic ion beams being replaced by cluster ion beams, such as Au_3_^+^ or Bi_3_^+^ (currently the most widely employed general imaging beam) [25, 26]. These cluster beams produce increased sensitivity while still being readily focusable to tens to hundreds of nanometres. Here, time-of-flight (ToF) analysers are normally used as they provide high sensitivity and parallel detection of all masses.

Further advances in molecular ion sensitivity were achieved with the use of polyatomic ion beams such as C_60_^+^ [27]. The most recent developments have focused around the implementation of gas cluster ion beams (GCIBs) that fire clusters of several thousand, super-cooled gas atoms/molecules at the sample [28]. Argon cluster ions, *e.g.*, Ar_4000_^+^, are most commonly used although CO_2_ clusters, *e.g.*, (CO_2_)_6k_^+^, have also been used [29, 30]. Recently published studies using GCIBs include investigations on infarcted mouse heart, aggressive basal cell carcinoma and rodent brain [31–33]. These beams have increased sensitivity to higher mass species; the Ar_4000_^+^ produces 30–50 times more signal from intact lipids including now being able to image gangliosides in brain. However, this has come at the loss of spatial resolution with typical ‘best’ spot sizes from GCIBs being around 1–5 μm. Improving the resolution of these beams is an active development area. The increased intact molecular signal afforded by these beams has created a closer synergy between the SIMS and MALDI approaches to mass spectrometric imaging. The majority of studies using GCIBs have focused on tissue imaging while C_60_ and Au/Bi_3_^+^ beams are used when sub-cellular resolution is required.

**Fig. 4:**
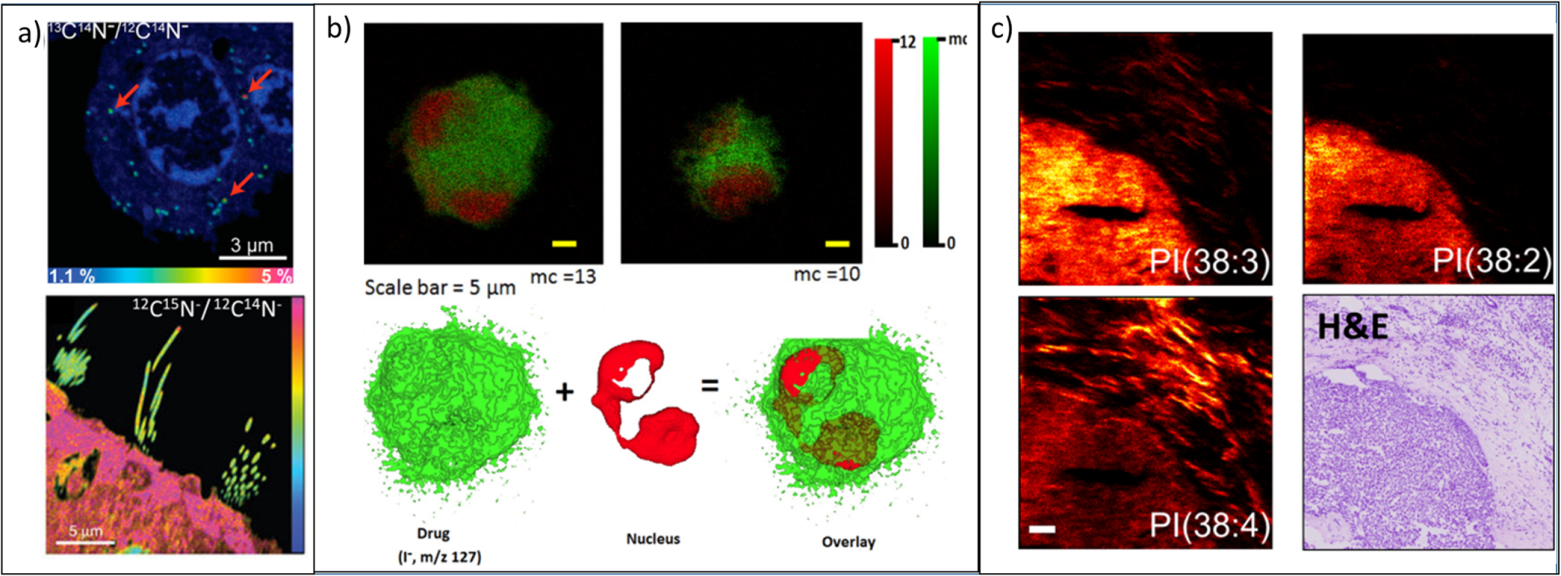
(**a**, upper) NanoSIMS image of the ^13^C^14^N^−^/^12^C^14^N^−^ ratio image reveals the dopamine enrichment in single vesicles in pheochromocytoma (PC12) cells. Red arrows highlight three examples of vesicles. Reproduced from [34]. (**a**, lower) NanoSIMS image of ^12^C^15^N^−^/^12^C^14^N^−^ ratio showing low incorporation of new proteins in stereocilia after feeding mice for 56 days with food containing ^15^N-leucine. Reproduced from [35]. (**b**, upper) ToF-SIMS images of NR8383 macrophage doped with desethylamiodarone (DEA) in negative ion. Snapshot images acquired using the Bi_3_^+^ ion beam for analysis and a GCIB for sample etching at different stages of the 3D image acquisition iodine, I^−^ (green), and the summed ion contribution of the nuclear-markers (red). (**b**, lower) 3D isosurface rendering of the doped cell. I^−^ is mapped in green and nuclear marker, HP_2_O_6_^−^, is mapped in red. Reproduced from [36]. (**c**) ToF-SIMS images of a human breast cancer biopsy using a (CO_2_)_6k_^+^ GCIB with H&E-stained image from a consecutive tissue slice. Three different phosphatidylinositol (PI) lipids distribute differently between the tumour and the inflammatory cells in the surrounding stroma. Reproduced from [37].

Fig. 4 illustrates the typical resolution and chemical information available using different SIMS methods. NanoSIMS images in Fig. 4a show isotope ratio images using the CN^−^ ion. In the first instance, C-13 is used as an isotopic label to localise dopamine within vesicles, and in the second example, N-15 is used to look at protein turnover in mouse stereociliae. ToF-SIMS images, in contrast, have lower spatial resolution but are still able to see large cellular components (*e.g.*, nucleus) by imaging small molecules or characteristic fragments of those molecules. 3D molecular imaging as shown in Fig. 4b is possible using polyatomic and gas cluster beams and has been applied to various cell and tissue types including mammalian cells, frog eggs, protozoa and rodent brain [36, 38–40]. In Fig. 4b, desethylamiodarone (DEA) is imaged using its I^−^ fragment along with characteristic signals known to be typical of the cell nucleus [41]. GCIBs facilitate the imaging of intact lipid species as Fig. 4c shows this in a breast cancer biopsy sample, with three lipids showing different distributions in tumour cells and inflammatory cells in the stroma. While intact lipids are now readily imaged, the spatial resolution in this case was set to 4 μm/pixel using an ion gun capable of 2–3 μm spot sizes. New gas cluster ion beam systems have been developed that can be focused to 1 μm spot sizes for sub-cellular imaging with early, unpublished data, which has been shown at recent conferences. The increased molecular signal from new GCIBs and the improvements in spatial resolution with MALDI have produced an overlap in the capabilities of the two techniques that has generated new questions of how best to use them for complementary analysis that are a current source of debate and investigation within the MSI community. One drawback of the GCIBs on conventional ToF-SIMS instruments is that it is difficult to achieve very short primary pulse widths that are normally required for good mass resolution. The latest generation SIMS instruments are optimised for exploiting the capabilities of these new beams and are operated with a (quasi-) continuous primary ion beam either by bunching the secondary ions or by using an Orbitrap analyser as a second mass analyser on a hybrid Orbitrap-ToF-SIMS instrument [42, 43].

Sample preparation for SIMS is often described as being simple or not required. However, while matrix addition is not required as in MALDI, maintaining sample integrity during the transfer to, and subsequent analysis in, a vacuum environment is critical. Fast freezing and analysing a sample at cryogenic temperature in the vacuum system is considered the gold standard. The advantage of this approach is that it provides the highest degree of confidence that the chemical distribution within the sample represents the ‘life-like’ state. This has been shown to produce improved imaging results for 3D sub-cellular imaging of cultured cells where the sample is gradually eroded by the ion beam to produce images such as the one in Fig. 4b and 2D imaging of tissue slices where migration of chemicals to the surface of the tissue during freeze drying can be a problem [44, 45]. This is in stark contrast to the MALDI experiment where relocation of the analyte for incorporation with the matrix is desirable. While treatment of the sample with exogenous compounds is not required, there can be benefits of preparation with reactive vapour [44, 46], the addition of a MALDI-type matrix [47, 48] or biochemical reactions on the surface of the sample [49].

### Imaging Mass Cytometry (IMC), a New Label-Based MSI to Follow Protein Markers at Cellular Level

**Fig. 5:**
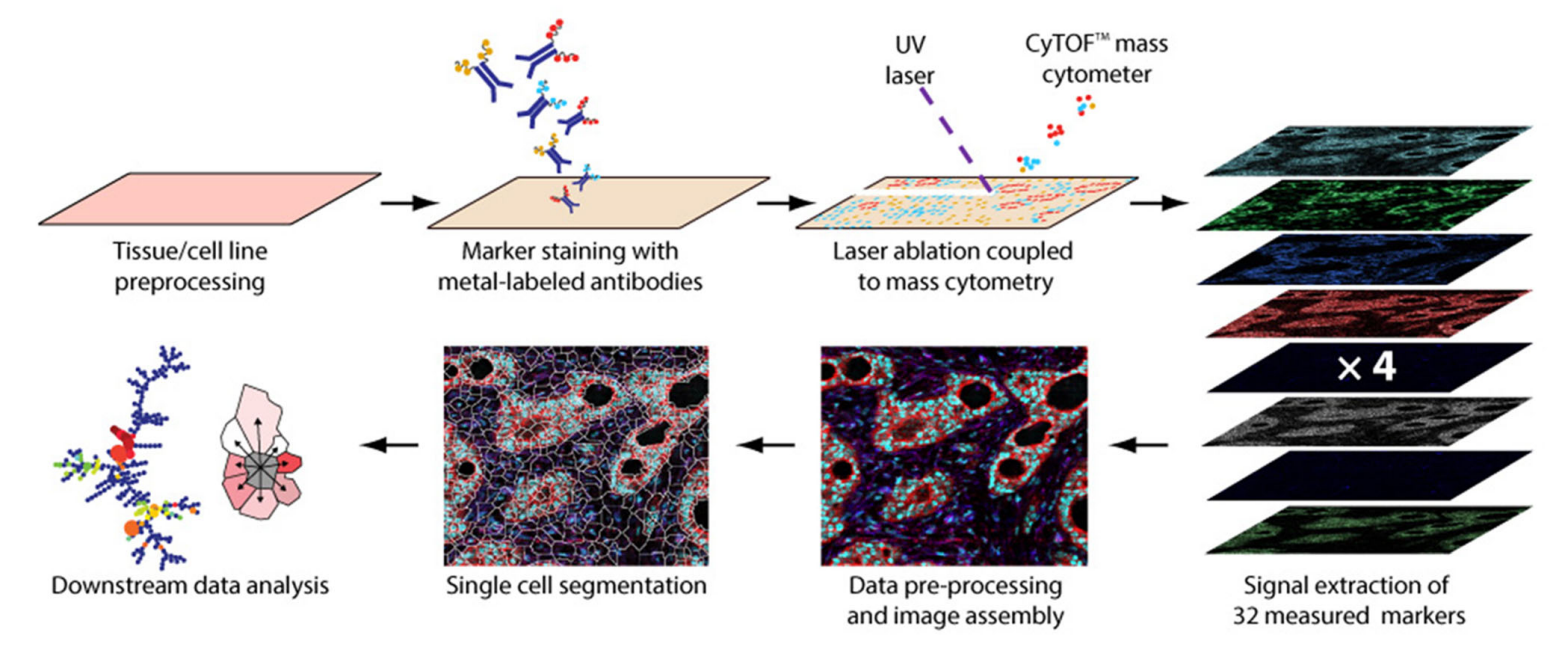
Imaging mass cytometry (IMC) workflow. Reproduced from [51].

IMC is the combination of MSI, flow cytometry and IHC enabling multiplexed and spatially resolved single-cell proteomic analysis [50]. The workflow of this recent technique is shown in Fig. 5.

Fresh frozen or FFPE tissue sections are stained using a cocktail of antigen-specific antibodies conjugated to different metal isotopes, allowing up to 32 different probes simultaneously. Unlike flow cytometry, IMC antibody labelling is not based on fluorophores but on metal tags reducing signal fading, spectral overlap or autofluorescence and increasing multiplexing capabilities, specificity and sensitivity. IMC uses an inductively coupled plasma (ICP) ion source with a high-energy UV laser combined with a ToF-MS instrument for fast elemental analysis [52]. The focused laser beam (1 μm) ablates the tissue surface following a virtual raster inducing the cleavage of the metal tags from the antibodies. Metal isotopes from a discrete position on tissue or pixel are then quantitatively analysed by the mass spectrometer based on their *m*/*z* values. Thus, metal localisation and concentration can be linked to their conjugated antibody target providing tissue/cells phenotyping information. It enables the simultaneous visualisation on the same tissue section of multiple markers from proteins and/or cells allowing a statistical data analysis such as cell segmentation/counting or cell neighbourhood/distances measurement. Only a few applications of IMC have been published so far, mostly reporting the novelty of the technique as well as the specificity of the instrumentation [50, 51, 53]. IMC is mainly applied for cancer research to better characterise and understand the tumour microenvironment (TME) which is of great importance for new therapeutic target discovery. It can also be employed in preclinical and clinical pharmaceutical research for the multiplexed spatial analysis of static and pharmacodynamic biomarkers.

**Fig. 6:**
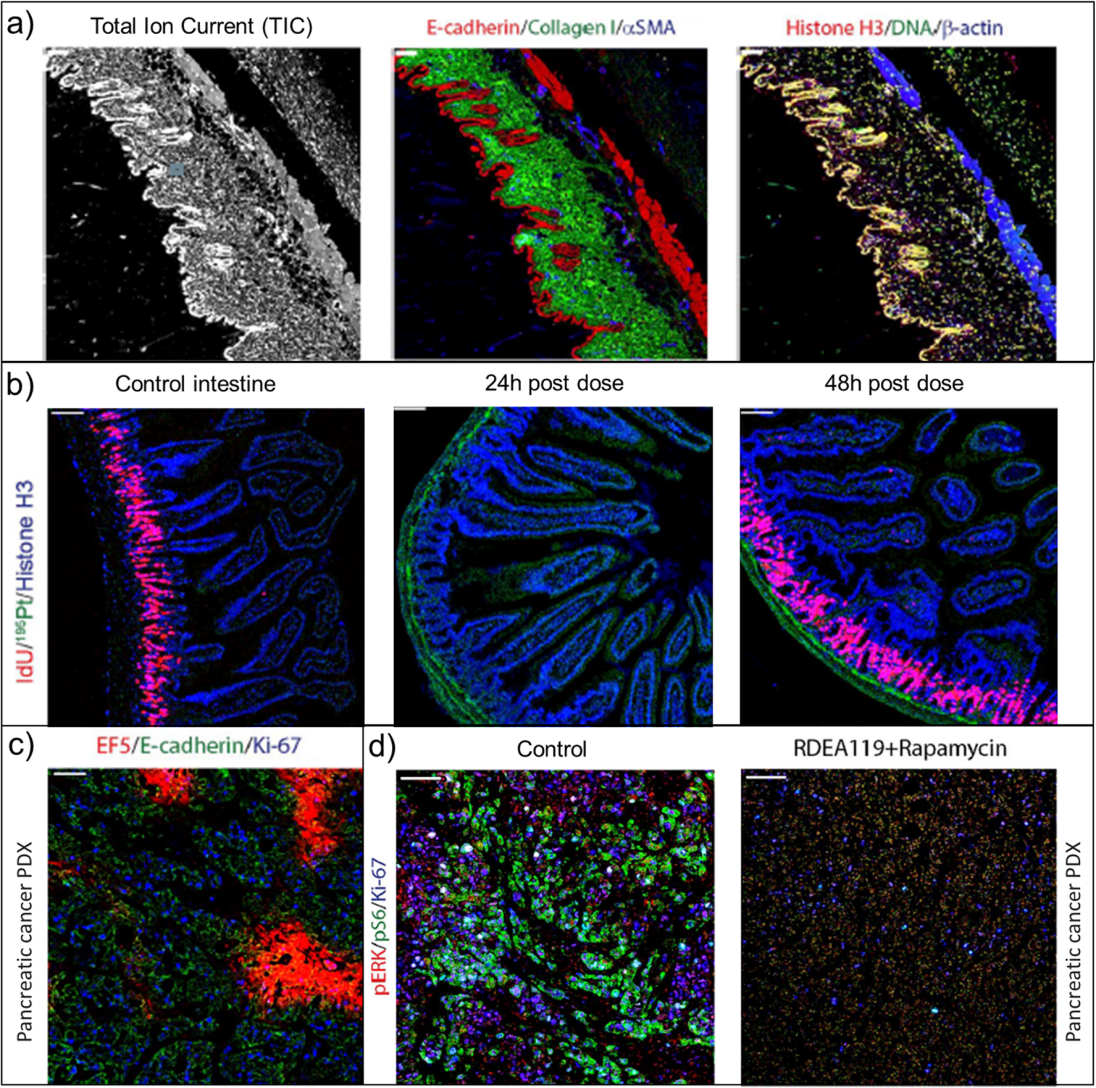
Selection of IMC images from various applications adapted from [53, 54]. (**a**) *Tissue architecture*: representative total ion current image (left) of mouse abdominal wall, overlay of three tissue type markers (middle) β-actin, histone H3 and iridium DNA intercalator, (right) E-cadherin, collagen and αSMA. (**b**) *Functional markers*: representative IdU, ^195^Pt and histone H3 images of control and cisplatin-dosed (4 mg/kg for 24 and 48 h) small mouse intestine. (**c**) *Tumour microenvironment (TME)*: pancreatic cancer patient–derived xenograft (PDX) assessment using EF5 tracer, E-cadherin and Ki-67. (**d**) *Target engagement*: control and dosed pancreatic cancer PDX tissue evaluation.

Various application examples are displayed in Fig. 6 [53, 54]. Anatomical features are highlighted based on the total ion current (TIC) image corresponding to extracted signal from measured markers as shown on the left panel of Fig. 6a. It is possible to orient the image and generate a spatial segmentation of the image. Specific cell clusters can be isolated from the whole image for further statistical analysis or to support histological evaluation. Middle and right panels of Fig. 6a show overlays of some of these markers obtained from mouse abdominal wall tissue. Epidermis is strongly stained for E-cadherin whereas collagen fibres (collagen I) are concentrated in the dermis layer. Vascularisation of tissue through blood vessel localisation can be visualised using αSMA (smooth muscle actin) marker. Lastly, anti-histone H3 and iridium-containing DNA intercalators describe nuclei (shaded yellow colour corresponding to the overlay of both markers) and are spatially correlated with epidermis.

Chang *et al*. reported the analysis of cisplatin using IMC to provide new insight on its pharmacological effects in the small intestine basal crypt cells [53]. Fig. 6b shows the distribution of major isotopes of platinum, Pt-195, in dosed mouse small intestine. Cisplatin appears to be mainly localised in the lamina propria and outer wall of the small intestine even at the earlier time point. The *in vivo* administered tracer, iododeoxyuridine (IdU), was used as a functional marker of cell cycle modulation. Fig. 6b shows an intense IdU uptake by the basal crypt cells of control intestine, which is greatly reduced 24 h after administration, but resumed after 48 h. This multiphasic IdU uptake suggests an adaptive response of cells to cisplatin enabling drug-induced damage to be repaired during a brief period of cell cycle arrest, and then resumption of normal activity. This indicates that cisplatin has a significant acute effect on the small intestinal mucosa in altering biochemical processes.

IMC also offers the opportunity to investigate the heterogeneity of the TME and understand cancer development and resistance thanks to disease-related probes. The biology of tumour hypoxia has been studied by Chang *et al*. using IMC [54] and is presented in Fig. 6c. The 2-nitroimidazole tracer EF5 revealed hypoxic regions within pancreatic cancer patient–derived xenografts (PDX) alongside a proliferation marker Ki-67 and a cancer cell marker E-cadherin. Distribution of hypoxia is anti-localised with proliferative cells showing the high degree of complexity of the TME. Fig. 6b shows IMC images from a control and a dosed pancreatic cancer PDX using a combination of two inhibitors targeting the ERK (RDEA119) and mTOR (rapamycin) pathways [54]. Their anti-cancer activity was evaluated by following the modulation of corresponding signal pathways by IMC after dosing. Phosphorylated ERK (pERK) and S6 ribosomal protein (pS6) appeared to be strongly decreased in the dosed sample indicating a strong suppression of these pathways. These studies highlight the ability of IMC to support efficacy and target engagement validation studies in drug discovery.

Based on these example applications, the potential of merging IMC with other modes of MSI is obvious. Conventional MSI is focused on small molecular species, drugs, metabolites or lipids with also the ability to detect most abundant peptides and proteins whereas IMC is dedicated to proteomic and cellular markers. Both techniques can be used on the same or adjacent tissue sections. Complementary information can be extracted from this multimodal setup to obtain a more complete picture of biological processes within tissue. Currently, however, there is no published example of MSI being combined with IMC.

Table 1 compares both techniques in terms of performance and limitations. The main differences are study design, throughput—either for the preparation of samples or their analysis—and dynamic range. IMC is always a targeted approach, meaning one has to select antigens/antibodies prior to the analysis. MSI has the strength of providing an untargeted approach as well as not relying on binding capabilities/specificities of antibodies (especially when it comes to post-translational modification or protein isoforms). In terms of analysis throughput, a first MSI step can be used to guide further IMC experiments on biologically relevant tissue sub-regions or to guide the selection of protein markers. Multimodal IMC/MSI experiments appear to be a powerful new research tool to understand tissue biology at many molecular layers and to provide an ‘omic’ systems-level analysis.

**Table 1:** MALDI and DESI-MSI *versus* IMC comparison

	DESI-MSI	MALDI-MSI	IMC
Spatial resolution (μm)	~ 50	5–10	1
Multiplexing	> *m*/*z* 1000 per sample	> *m*/*z* 1000 per sample	30 protein markers per sample
Target size (*m*/*z*)	50–1000	Unlimited (untargeted)	Unlimited
Molecular classes	Drugs, metabolites, lipids	Drugs, metabolites, lipids, peptides, proteins	Limited to targeted proteins
Quantitation	Possible		
Throughput	< 5 min/mm^2^	< 5 min/mm^2^	2 h/mm^2^
Sample preparation (other than tissue sectioning)	0 min	0–1 h (+ overnight in case of on-tissue digestion)	3–5 h (+ overnight)
Output	Proprietary or Imzml file	Proprietary or Imzml file	MCD and text file

### Data Interpretation and Integration Using Dedicated Computational Tools

MSI data may be considered as a ‘datacube’ comprising a series of mass spectra, from discrete *x*,*y* and sometimes *x*,*y*,*z* locations. To visualise the distribution of a given ion of interest, the intensity of the ion at each spectrum is plotted to provide a 2D or 3D image. Colour schemes for these images should be carefully selected as they affect the perceived structure within the data and therefore the resulting interpretation [55]. Given the size and complexity of MSI data, producing ion images from raw data and reviewing the distribution of ions of interest is a time-consuming process. Furthermore, sophisticated methods are required to determine and review trends, associations and correlations between groups of molecules detected from different regions. It is becoming increasingly common to employ computational techniques to objectively identify trends within the data and to build models for tissue/disease classification.

Processing of MSI data includes pre-processing (the set of processes that correct for variations in the data, experimental artefacts and noise, and result in peak picked data) and post-processing (statistical analysis performed on the peak picked data to identify trends within the data). Standard pre-processing steps include generation of a consistent *m*/*z* axis, peak detection, dead-time correction, smoothing, baseline correction, normalisation and peak alignment [56]. Selection of pre-processing stages, *e.g.*, baseline subtraction, may not be universally suitable for data collected using different instruments. A wide range of vendor-specific and open-source packages are now available. For some tasks, a consensus on the best approach is not clear. For example, several normalisation methods have been proposed, such as median, internal standard, total ion current (TIC), root mean square (RMS), ‘median of informative peaks’ [51] and variance stabilising. An ongoing challenge for the MS community is evaluation of new methods and the absence of a ‘ground truth’. Model datasets [52] and methods for generating synthetic data are emerging as valuable resources*.*

A further difficulty in ‘making sense’ of vast MSI data is the challenge associated with gaining a reliable identification and annotation of molecules detected. Public databases, *e.g.*, lipidMAPS or HMDB, provide thousands of candidate identities against which MS and MSI data can be searched. A major challenge is that unlike other -omics strategies, for MSI the separation of extracted molecules by chromatography is not feasible. For data collected using high-mass-resolving-power instruments, an online annotation engine, METASPACE, provides an annotation tool for MS imaging data [57]. Accuracy of metabolite identification is improved by use of a false discovery rate algorithm which takes into account the isotope pattern and ion images of candidate molecules.

Post-processing of mass spectrometry imaging data often involves univariate analysis [58], multivariate analysis [59] or the application of clustering [60] or classification [61] algorithms. Many techniques currently exist in the literature [54]; however, only a few are implemented in available software and even fewer are available together in software capable of handling data from multiple platforms [58, 59]. The usefulness of using more than one method for data analysis, *e.g.*, PCA, NMF and PLSA, was illustrated in the case of exploring tumour heterogeneity [55]. Clustering or segmentation methods, which aim to group similar spectra together based on a distance metric, are also used for these purposes. In early studies, *k-*means and agglomerative hierarchical clustering were used to segment anatomies from rat tissue [62]. Since then, the more efficient bisecting *k-*means hierarchical clustering has been used to cluster large MSI datasets including 3D MSI images [63]. Alexandrov *et al*. introduced a method to overcome the pixel-to-pixel variability observed in MSI data and provide a more accurate segmentation of coronal mouse brain images [64]. Recently, a graph-based algorithm with a two-phase sampling method was presented by Dexter *et al*., proving suitable for both large and noisy datasets.

Several groups have shown how t-distributed stochastic neighbour embedding (t-SNE) algorithm can provide powerful visualisations of MSI data. t-SNE is an unsupervised dimensionality reduction technique initially developed for visualisation of high dimensional data in two or three dimensions [65]. The algorithm embeds high dimensional data preserving the local distance between data points into a lower dimension. This method provides a powerful embedding of the data making it extremely useful for visualisation purposes. When first applied to MSI data [66], the authors demonstrated enhanced embedding when combined with signal normalisation. For MSI, t-SNE is able to embed the full mass spectrum at each pixel (high dimensional space) to three dimensions per pixel enabling the data to be visualised as a single RGB image. The ability of t-SNE for dimensionality reduction of MSI data has been used in registration of MS images to reference material (brain atlas and histology) [67], classification tasks in tumours [68] and assessing heterogeneity in cancer [69].

To date, there have been relatively few performance metrics proposed for assessing the performance of clustering or segmentation routines. This is due to the lack of a ground truth, as mentioned above. New methods and metrics to objectively evaluate the success of these methods are required. As we move into using these methods for grouping data into biologically relevant categories (*e.g.*, tumour phenotypes within and between different patients), approaches for internal and external validation of the classification is needed.

With ongoing improvements in instrument performance, the size and complexity of MSI data continues to grow. Furthermore, improved performance increases usefulness and uptake of the techniques. As widespread application of MSI continues, new, more efficient methods for data reduction and data processing will be required.

### Correlative MSI and Other Imaging Modalities—Towards an Integration of *Ex Vivo* and *In Vivo* Data

One of the current focuses of the MSI community is the integration of specific localised molecular information with other imaging modalities to gain better insights in biological tissue. Roddy *et al*. showed that bright-field, scanning ion, fluorescence microscopy and TOF-SIMS imaging provide deep understanding of the functional role of specific lipids in the function of cellular membranes [70]. Vollnhals *et al*. recently described approaches (*i.e.*, Laplacian pyramid fusion and hybrid fusion) for image fusion in the context of combining the inherently lower-resolution chemical images obtained using SIMS with the high-resolution ultrastructural images obtained using electron microscopy (EM). LPF was found to overcome limitations such as intensity saturation which can be encountered when dealing with contrast mismatch in input images [71].

MSI combined with fluorescence was used to track endogenous markers such as lipofuscin—an ageing marker in the retinal pigment epithelium (RPE) associated with the development of age-related macular degeneration [72]. Although lipofuscin is fluorescent, MSI enabled unambiguous identification of the molecular substructure of this clinically relevant diagnostic marker. MALDI-MSI was employed to investigate the spatial distribution of the drug and its metabolites after local administration, and quantify drug levels in tumour and liver tissue over time [73]. This study demonstrated the strength of MSI to differentiate a primary drug from its metabolites, which is not possible with fluorescence in this particular case. Correlated MSI and confocal Raman microscopy is also powerful for the study of three-dimensional cell culture sections [74].

The recent combination of MSI data with magnetic resonance imaging (MRI) constitutes a major step towards bridging the gap between the *ex vivo* and *in vivo* imaging. Together, MRI and MSI may lead to a powerful combination and open new doors to connect biomolecular pathways with human diseases and metabolic disorders. This will potentially lead to a better understanding of molecular pathways and basic biological processes, support diagnosis in early stages of disease, improve assessment of treatment effectiveness and help in the development of new treatments. MRI is an established biomedical imaging technique to study the anatomy and physiological processes of the body in both health and disease states. It provides high-resolution tomographic images with excellent soft tissue contrast. With MRI, it is possible to study dynamic organ function, such as movement of the heart and blood flow. It is also possible to follow a disease over time, even before clinical symptoms occur. Conversely, MSI offers the opportunity to translate the molecular changes in cells to disease processes observed in patients.

MSI is paving a path of establishment amongst the molecular imaging techniques. The strength of MSI is in enabling the imaging of thousands of molecules at once without the need of prior knowledge of a sample and without labelling. It has already been demonstrated that its ability to target specific markers—such as contrasts agents used for MRI—can provide useful complementary information to *in vivo* MRI. In 2015, Tata *et al*. employed DESI-MSI to map intra-tumour heterogeneity such as vasculature and margins by targeting a MRI contrast agent (gadoteridol) that accumulates in breast tumour tissue [75]. With this approach, it was possible to characterise molecular intra-tumoural heterogeneity and precisely localise tumour margins. In this case, one of the advantages of monitoring a specific agent is the possibility to identify tumour margins without building an extensive tissue-specific database beforehand [76]. This is of prime importance during intraoperative tissue assessment. DESI-MSI has also enabled imaging of the flux dynamics of a contrast agent through mouse kidneys to demonstrate its excretion.

The group of Agar *et al*. described another demonstration of the relevance of MSI-based approaches complementing MRI in intraoperative decision-making [77]. In their innovative Advanced Multimodality Image Guided Operating (AMIGO) suite, the group used DESI-MSI to monitor an onco-metabolite (2-hydroxyglutarate; 2-HG) to guide brain tumour surgery. MSI analysis was performed in surgically resected specimens during the surgery and enabled tissue and tumour margin assessments within a few minutes. This can take up to 30 min in a routine clinical workflow where a pathologist assesses stained sections outside of the theatre. Using the AMIGO approach, the targeted metabolite signal was mapped onto 3D MRI reconstructions of tumours allowing the integration of molecular and radiological information for enhanced clinical decision-making [78]. Another approach combined molecular profiling and multivariate statistical analysis to build classification models. In this way, Eberlin *et al*. pushed the limits of MSI not only to delineate tumour boundaries but to facilitate quasi real-time tumour classification and staging [79] discriminating gliomas and meningiomas (based on 36 glioma and 19 meningioma samples).

MALDI-MSI has also been successfully applied to monitoring gadolinium (III)-based contrast agents known to improve the sensitivity and specificity of MRI in a study of cardiovascular disease [80]. Here, Aichler *et al*. used MSI to evaluate the specificity of the MRI probe in a mouse model of myocardial infarction and to perform *in situ* relative quantification of several contrast agents including gadofluorine M—which is still challenging with MRI—to gain insights into the dynamics of the probe over time.

Recently, laser ablation inductively coupled plasma MS (LA-ICP-MS) has been used to observe cobalt levels within tumour spheroids (representing 1.5 × 10^4^ cells) and characterise tumour microenvironment [81]. Cobalt bioreductive pro-drugs selectively release toxic payloads upon reduction in hypoxic cells and have shown great promise as anti-cancer agents. However, their response in the tumour microenvironment is not well understood. As cobalt complex can be used as contrast agent, MRI was used to monitor changes in water signal induced by reduction from diamagnetic Co (III) to paramagnetic Co (II). This exemplified the relevance of correlating MRI/MSI to better understand drugs promoted by hypoxia and more generally for monitoring paramagnetic metal-based therapies.

**Fig. 7:**
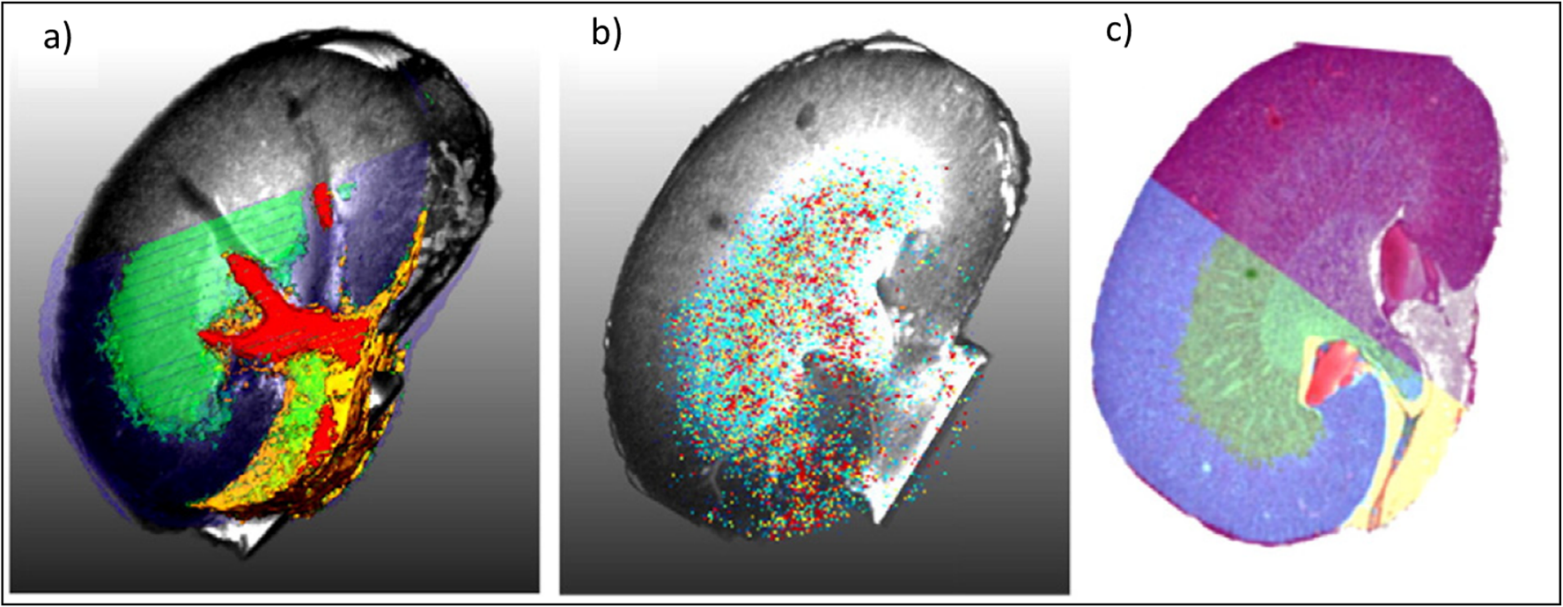
Example of a multimodal study combining MRI, 3D-MALDI-MSI and histology (H&E). (**a**) Semi-transparent MRI volume rendering overlaid with the semi-transparent clusters from 3D-MALDI-MSI data showing the renal cortex (blue), the medulla (green) and pelvis (red). (**b**) Overlay of the 3D molecular map of ion species at *m*/*z* 4808 with MRI volume rendering. (**c**) Overlay of H&E-stained section from the inner kidney with 3D segmentation map of the same tissue. Reproduced from [82].

To co-register and fuse the imaging data and information generated for large datasets from different imaging modalities, sophisticated image processing and data handling routines are required. Image fusion refers to the generation of a single image from several source images and aims to provide a more accurate description of the sample or combine information towards a particular human or machine perception task. As such, Van de Plas *et al*. [83] described such ‘predictive imaging modality’ by fusing MSI and microscopy, which even allowed to predict ion distribution in tissues where the MSI was not acquired, based on high-resolution optical microscopy image. This remains challenging and complicates the extraction of relevant information. If the speed of acquisition inherent to mass spectrometry instrumentation has been one of its main limitations, it is noteworthy that the latest instrumental developments have considerably improved throughput to clinically relevant time scales [84]. A few studies have focused on the integration of MSI and MRI datasets [82, 85–87]. After fusion of reconstructed scan images and MRI data, the slice-related coordinates of the mass spectra were propagated into 3D space. Once image registration of scan images and histological H&E-stained images is achieved, anatomical information from histology can be linked to the mass spectra from MALDI-MSI as shown in Fig. 7.

To speed up and automate feature extraction based on an anatomy-driven strategy, algorithms have been developed to integrate MSI measurements with anatomical atlases such as the Allen Mouse Brain Atlas as described by Verbeeck *et al*. [88–90]. MSI data are spatially registered with anatomical data using three individual registration steps: (1) rigid registration of MSI data to experiment histology, (2) rigid registration of anatomical data to reference histology and (3) non-rigid registration of experiment histology to reference histology. This workflow allowed rapid correlation between molecular information and anatomical annotation [90]. This was further extended to the so-called Scalable Brain Atlases, which are mainly based on MRI data. The approach was described to be robust enough to account for biological variation occurring between different animals—as would always be the case when co-registering one’s own experimental data with a well-documented atlas. In addition, this approach allows for direct integration of MSI with MRI data without using intermediate optical images (*i.e.*, pre-MSI optically scanned images or H&E-stained sections).

## Conclusions

Latest developments in overall analytical workflow and instrumentation have contributed to render MSI a unique tool in biomedical research. The MSI community is continuously improving its sample preparation processes to produce more robust protocols and get the most of the tissues analysed. MSI is now able to detect and characterise a wide variety of biomolecules, ranging from small metabolites to large (intact) proteins and antibodies, and including lipids, peptides (endogenous and digested) and glycans. Innovation in instrumentation has considerably strengthened MSI, which can now provide unique molecular information at unprecedented combined spatial resolution and sensitivity. It is possible to image potential markers down to cellular resolution or to analyse large patient cohorts with a speed matching clinical needs. For those reasons, MSI is employed more and more in clinical research and is being integrated in the exciting field of molecular pathology to complement routine histopathology tissue examination. As such, MSI enables an inimitable characterisation of tissue microenvironments and provides crucial information linking tissue morphology to its (molecular) physiology.

One of the current big challenges the MSI community is facing remains the management and interpretation of big data. The community is investing a lot in developing innovative processing software to get the most out of the data. Processing is not only limited to finding algorithms for accurate co-registration but for extracting meaningful data that can be linked to translational patient characteristics such as disease-free survival, recurrence or response to a therapeutic treatment.

Another challenge is the integration of molecular information provided by MSI with *in vivo* imaging modalities such as PET or MRI. Early stage publications have indicated great translational potential from these multimodal investigations and algorithms have been developed to tackle some of the limitations related to co-registration. However, these workflows are not yet trivial. Promising developments are ongoing and major steps are being made towards bridging the gap between *ex vivo* and *in vivo* imaging worlds which could greatly accelerate integration of MSI into clinical care.
